# 

**Published:** 1906-08-18

**Authors:** 


CONTENTS.

**The Natural History Museum** - 339

**Longevity** - 340

**Annotations** - 341

**British Methods and Men of Science** - 342

**Hospital Clinics—**

Chronic Suppuration in the Nasal Accessory Sinuses and its Treatment - 343

**Progress in Medicine and Surgery—**

Abdominal Epilepsy - 347

Diseases of Abdomen - 343

**Special Analytical and Health Commission on Tinned and Preserved Food** - 349

**The Book World of Medicine and Science** - 351

**New Appliances and Things Medical** - 352

**Hospital Administration—**

British and American Hospitals - 353

Studies in Poor-law Expenditure - 355

The Plane Tree of Hippocrates - 355

Structural and Practical Departments - 356

*NURSING SECTION.*

**Notes on News from the Nursing World—**

The Question of Previous Training—Midwifery in Poor-law Infirmaries—An Incident which ought to be Impossible—Nurses and Tram Fares—Insufficient Staff at Exeter Infirmary—The Injured at the Handcross Accident—A Pin as a Credential—Central Midwives Board Examination—Opening of a New Tennis-lawn at the Birmingham City Hospital—District Nursing in Ireland—A Notable Institution at Sydney—Progress at Grantham—Short Items - 281–283

**The Nursing Outlook** - 284

**The Care and Nursing of the Insane** - 285

**The Nurses' Clinic** - 286

**Incidents in a Nurse's Life** - 287

**The Nurses of the General Hospital, Birmingham** 287

**Isthmian Canal Service in Panama** - 289

**Our Summer Holiday** - 290

**Bedside Words** - 291

**Saving herself Trouble** - 291

**Everybody's Opinion** - 292

**Appointments** - 292

**The Nurses' Bookshelf** - 293

